# Severe Quantitative Scale of Acanthosis Nigricans in Neck is Associated with Abdominal Obesity, HOMA-IR, and Hyperlipidemia in Obese Children from Mexico City: A Cross-Sectional Study

**DOI:** 10.1155/2022/2906189

**Published:** 2022-03-28

**Authors:** Ana I. Burguete-García, Alan Gilberto Ramírez Valverde, Meztli Espinoza-León, Isaac Sánchez Vázquez, Evelyn Yazmín Estrada Ramírez, Itzel Maldonado-López, Alfredo Lagunas Martínez, Cinthya Estefhany Diaz Benítez, Roberto Karam Araujo, Diana Fernández-Madinaveitia, Adriana E. Anides Fonseca, Miguel Cruz, José de Jesús Peralta Romero

**Affiliations:** ^1^Departamento de Infecciones Crónicas y Cáncer, Instituto Nacional de Salud Pública, Cuernavaca, MOR, Mexico; ^2^Unidad de Investigación Médica en Bioquímica, Unidad Médica de Alta Especialidad “Dr. Bernardo Sepúlveda”, Centro Médico Nacional Siglo XXI, Instituto Mexicano del Seguro Social, Ciudad de México, Mexico; ^3^Escuela Nacional de Medicina y Homeopatía, Instituto Politécnico Nacional, Av. Guillermo Massieu Helguera 239, La Escalera, Gustavo A. Madero, Mexico; ^4^Departamento de Prestaciones Económicas y Sociales Del Instituto Mexicano del Seguro Social, Avenida de La República, No. 154, Colonia Tabacalera, Ciudad de México, Mexico; ^5^Unidad de Dermatología, Unidad Médica de Alta Especialidad “Dr. Bernardo Sepúlveda”, Centro Médico Nacional Siglo XXI, Instituto Méxicano del Seguro Social, Ciudad de México, Mexico

## Abstract

**Background:**

Acanthosis nigricans (AN) is a clinical sign that commonly occurs in obesity; however, its specificity and sensitivity have been controversial. It is unknown if AN severity degree can be a useful marker for cardiometabolic disorders screening. We suggest that the stratified analysis of AN severity degree in neck by Burke's scale could be a useful tool in the screening of cardiometabolic alterations in obese children.

**Objective:**

The aim of this study was the association of AN severity degree in neck by Burke's scale with anthropometric, biochemical, and inflammatory parameters in obese school-age children from Mexico City.

**Methods:**

A cross-sectional study was conducted, including 95 obese school-age children stratified by AN severity degree in neck by Burke's scale. Anthropometric and fasting biochemical measurements were determined. Variables were compared by *x*^2^ test for frequencies and one-way ANOVA with Bonferroni posttest for continuous variables. Linear regression analysis adjusted by gender, BMI, and age was performed to evaluate the association between AN severity degree and cardiometabolic alterations. Statistical significance was set at *p* < 0.05.

**Results:**

As AN severity degree in neck by Burke's scale increased, diastolic blood pressure (*p*=0.001) and triglycerides (*p*=0.02) significantly increased and adiponectin significantly decreased (*p*=0.02). Positive associations between grade 3 AN and waist circumference, HOMA-IR, triglycerides, total cholesterol, and LDL cholesterol were observed.

**Conclusion:**

Our findings could be used to identify an easier clinical tool to prevent obesity progression and its complications in pediatrics. There are no similar studies.

## 1. Introduction

Obesity is an important public health issue in the world. In Mexico, the combined prevalence of overweight and obesity in the school-age population was 34.4% in 2012 and 32.2% in 2016, and high estimates represent an urgency in the care and prevention of complications from the early stages of life [1]. Despite the declining trend, the prevalence of overweight and obesity likely did not decrease as the prevalence in rural areas increased [1, 2]. Studies have suggested that up to 80% of overweight children will become obese adults [3]. Obesity is associated with dyslipidemia, insulin resistance (IR), and glucose intolerance (GI), which are the main risk factors for the development of type 2 diabetes and cardiovascular diseases. One of the most deleterious metabolic derangements of obesity is dyslipidemia, both are frequently associated, because there is some phenotype of dyslipidemia when the body mass index is higher. Dyslipidemia is a multifactorial condition that can occur in normal-weight and obese patients; however, obese individuals exhibit a lipid profile known as atherogenic dyslipidemia, characterized by increased concentrations of triglycerides, total cholesterol, low-density lipoprotein cholesterol (LDL-C), and decreased concentrations of high-density lipoprotein cholesterol (HDL-C), considered as a risk marker for metabolic syndrome and diseases cardiovascular. There is still a long way to go to elucidate the complex pathogenesis of obesity dyslipidemia, but it has been observed that obesity mediated by an increase in fatty acid intake leads to dyslipidemia, leading to the development of IR in peripheral tissues and intravascular lipolysis [4] and enhancing vascular and liver damage to develop at an early age in obese children [5]. Specific biochemical and inflammatory markers indicate the onset and severity of obesity, including adipokines and the homeostatic model assessment for insulin resistance (HOMA-IR); however, not all medical services have access to these tests. Therefore, validating and stratifying phenotypic characteristics as clinical markers associated with metabolic alterations due to obesity are necessary, with the intention of being used in primary prevention.

Acanthosis nigricans (AN) is recognized as the most frequent dermatosis in obesity. Clinically, AN lesions are described as thickened, symmetrical grayish-brown hyperpigmentation plaques. Histopathologically, papillomatosis and hyperkeratosis are observed. AN is often found in folds and areas of flexion and is most frequently located in the neck [6]. AN is a clinical sign that has been associated with obesity, IR [7–10], hormonal disorders [11], lipoinflammation mediated by leptin and adiponectin [12], the use of certain medications and supplements, and some types of cancer. AN presence in obesity is often described by its severity and the patient's family history and race [13, 14]. The association of AN with alterations described in some populations is usually controversial because of the difficulty of establishing the sensitivity and specificity due to ethnicity and phototype [15–17], rendering incidence and prevalence studies difficult in different populations including children [18–22].

Different epidemiological studies have reported AN incidence of approximately 7% in general population, but it can be present in up to 74% of obese people [23].

Few AN prevalence studies in school-age children have been reported; therefore, AN should not be underestimated, especially in obese children, who are vulnerable to cardiometabolic disturbances [24]. However, there are still some uncertainties and conflicts to determine if AN can be of clinical utility in children, due to AN being a dermatological lesion with variable frequency that usually appears at birth, in school age or adolescence. On rare occasions, AN may be associated with a generalized affectation, its presence in children should not necessarily be considered as pathological [25, 26], and its sensitivity as a clinical tool associated with cardiometabolic alterations in children could be controversial; however, its study becomes important due to AN frequency is higher in obese children.

While several classification systems can be used to diagnose AN [13, 27]. Burke's scale, which was developed in a Mexican American population, is the most used system. Burke's scale classifies AN according to the severity on a scale of 0–4 based on the number of affected areas [28]. Neck is the most accessible and common anatomical region for its study. Few studies in Mexican children have described the association of the presence of AN with anthropometric, biochemical, and inflammatory parameters [29, 30]; however, no quantification and correlation of the severity of AN with cardiometabolic alterations at different sites are provided. Currently, it's unknown if AN severity degree in neck by Burke's scale can be a useful marker in obese children for screening for cardiometabolic alterations. Stratified analysis of AN severity degree in neck by Burke's scale could provide evidence of the sensitivity and specificity in screening. The aim of this study was to determine the association of AN severity degree in neck by Burke's scale with anthropometric, biochemical, and inflammatory parameters in obese, school-age children from Mexico City. Our findings could suggest in the future the clinical use of AN severity degree in neck by Burke's scale as an easily useful tool in screening for the progression of obesity disorders in pediatric patients.

## 2. Material and Methods

### 2.1. Study Design and Population

This cross-sectional study was conducted between February and June 2016. Participants were collected using a convenience sampling method by nonprobabilistic consecutive selection, out of a total of 1,500 children registers from a database. We included 95 school-age children with obesity diagnosis from 4 recreational and social centers in Mexico City (north, south, east, and west), Instituto Mexicano del Seguro Social (IMSS) insured, who reported anthropometric and biochemical parameters, blood pressure, HOMA-IR, adiponectin, leptin, and AN severity degree in neck by Burke's scale registered in the database. The sample was stratified into 5 groups according to AN severity degree in neck by Burke's scale on a 0–4 level [28].

Patients with a history or current diagnosis of any type of cancer, thyroid disorders, psoriasis, atopic dermatitis, autoimmune diseases, endocrinological diseases, liver disease, users of supplements, antioxidants, steroids, antihistamines or diet, physical activity, or under pharmacological weight loss treatment were excluded. Children with BMI ≥98th percentile were also excluded.

### 2.2. Anthropometric Profile

All participants were weighed using a digital scale (Seca, Hamburg, Germany), and height was measured using a portable stadiometer (Seca). Waist circumference (WC) of each participant was measured at the midpoint between the lowest rib and the iliac crest after a normal exhalation in the standing position. Obesity was determined using the calculated body mass index (BMI) and classified according to the standards reported by the Center for Disease Control (Atlanta, GA, USA) in 2000 (obesity ≥95th percentile of BMI) [31].

### 2.3. Blood Pressure

Blood pressure (BP) was measured using a mercurial sphygmomanometer (ALPK2, Tokyo, Japan) with the appropriate cuff size for arm length following the North American Task Force 2004 Guidelines. The BP was measured in the right arm with the participant in a sitting position. Two BP measurements were obtained with a resting time of 5 min between each measurement. The mean of the two measurements was used in the analyses [32].

### 2.4. Acanthosis Nigricans

Burke's scale was used to determine the severity of AN in the neck. When AN was not detectable on close inspection, the severity was considered grade 0. Grade 1 severity was defined as AN is clearly present on close visual inspection but not visible to the casual observer and not measurable. Grade 2 severity was defined as AN limited to the base of the skull that did not extend to the lateral margins of the neck (typically <7.62 cm in breadth). Grade 3 severity was defined as AN extending to the lateral margins of the neck and the posterior border of the sternocleidomastoid (typically spanning 7.62–15.24 cm) that was not visible when the participant was observed from the front. Grade 4 severity was defined as AN extending anteriorly (typically >15.24 cm in breadth) that was visible when the participant was observed from the front [28].

### 2.5. Biochemical Profile

The following biochemical data were recorded: fasting glucose (mg/dL), insulin (µU/ml), total cholesterol (mg/dL), LDL-C (mg/dL), HDL-C (mg/dL), and triglycerides (mg/dL). These parameters were measured using the ILab 650 Clinical Chemistry System (Instrumentation Laboratory, Barcelona Spain) and Advia Centaur CP Immunoassay System (Siemens, Berlin, Germany). Adiponectin and leptin were also included and measured using the ELISA method (Human ELISA Kit, Invitrogen, Thermo Fisher Scientific, California, USA). Insulin sensitivity was calculated by the Homeostatic Model of Insulin Resistance (HOMA-IR) using the following equation: [(fasting glucose (mg/dL)) (fasting insulin (*μ*U/mL))/405] [33, 34].

### 2.6. Ethical Considerations

This study was approved by the Research and Bioethics Committee of the IMSS (registration number R-2012-785–071). Written informed consent was obtained from the parents of each participant prior to the study.

### 2.7. Statistical Analysis

Variables are reported as means and standard deviations, as well as percentages, and the normality distribution was evaluated by the Shapiro–Wilk test. Variables were compared using a chi-square test for frequencies and one-way ANOVA with Bonferroni posttest for continuous variables to assess the significance of differences in Burke's scale 0 versus 1, 2, 3, and 4. To evaluate the association of the severity of AN in the neck with WC, HOMA-IR, and the lipid profile, a linear regression analysis adjusted by sex, BMI, and age was performed.

History of type 2 diabetes, overweight/obesity, age, and sex were analyzed first and variables that resulted in <10% change were not included in the subsequent analyses. Each covariate and its effect on the estimator were evaluated, and the final model included overweight or obesity, age, and sex. All analyses were conducted using Stata, SE v11.0 (StataCorp, US) and Epi Info^TM^ 3.3.2 (CDC, Atlanta, Georgia, US) software. Statistical significance was set at *p* < 0.05 [31].

## 3. Results

A total of 95 children were analyzed. The participants' characteristics are listed in Table 1. The presence of AN in the neck in obese children was not significantly different between participants with a family history of obesity and diabetes and those without. Diastolic BP (*p*=0.001) and triglycerides (*p*=0.02) were significantly increased in the groups as the severity of AN increased. Adiponectin concentration significantly decreased (*p*=0.02) as AN severity degree in neck by Burke's scale increased. BMI, WC, insulin, HOMA-IR, total cholesterol, LDL-C, and leptin increased as the severity of AN increased and the glucose and HDL-C decreased as the severity of AN increased, though the differences between the groups were not significant.

Linear regression analysis was performed to evaluate the association of AN severity degree in neck by Burke's scale with WC, HOMA-IR, and the lipid profile is listed in Table 2.

Our results showed that grade 3 of AN severity degree in neck by Burke's scale was associated with WC (Coef. = 5.49, Std. Err. 2.31, CI: 0.88; 10.08, *p*=0.02), HOMA-IR (Coef. = 1.43, Std. Err. 0.58, CI: 0.27; 2.59, *p*=0.01), triglycerides (Coef. = 44.20, Std. Err. 16.57, CI: 11.24; 77.16, *p*=0.009), total cholesterol (Coef. = 29.51, Std. Err. 12.28, CI: 4.60; 54.42, *p*=0.02), and LDL-C (Coef. = 26.94, Std. Err. 10.10, CI: 6.85; 47.03, *p*=0.009).

## 4. Discussion

We report that grade 3 of AN severity degree in neck by Burke's scale is associated with increased WC, triglycerides, LDL-C, total cholesterol, and HOMA-IR. The results could be of clinical utility as a screening method in the early stages of life, and we observe that most obese children have metabolic disorders related to high cardiovascular risk in the future. It has been recognized that AN is the most frequent dermatosis in obesity [35], and its presence has been reported to be directly proportional to the degree of fatness [36]. Also, the prevalence of AN in overweight or obese children has been reported as up to 60% [37]. AN lesions are easily recognized via an inspection of the skin and should be carefully monitored in obese patients. No study has described whether AN severity degree in neck by Burke's scale could be a clinical indicator associated with cardiometabolic alterations in school-age children. Thus, our research showed a potential use for primary prevention.

In this study, 74/95 (77.87%) obese, school-aged participants presented AN in the neck. Among the participants with a family history of type 2 diabetes, 66.66% had ≥ grade 1 AN in this study, which is higher than that previously reported in the San Antonio Family Diabetes Study [28], which found that 41.1% of participants with a family history of type 2 diabetes had ≥ grade 1 AN, which correlated with fasting insulin and BMI.

It has been reported that up to 30% of obese patients are metabolically healthy, have insulin sensitivity like healthy individuals, normal weight, decreased visceral fat, and lower cardiometabolic risk factors [38]; however, 74% of obese American adults have AN and increased severity of obesity and insulin [18]. Therefore, we assume that the high prevalence of AN in our study may be attributed to the severity of BMI, hyperinsulinism, and IR. These reports are consistent with our results regarding obese children. Biochemical markers [39], heritage, and environment are risk factors for type 2 diabetes even in pediatric patients [40, 41].

In this study, positive associations between the presence of AN and variables associated with obesity were observed, which is consistent with previous studies that reported dyslipidemia and IR in school-age children in Mexico [29, 30]. Compared to other studies, our work suggests that AN severity degree in neck by Burke's scale is mainly associated with IR and hypertriglyceridemia, and clinical alterations can lead to vascular alterations even in children.

Martínez-Rojano et al. reported increased systolic and diastolic BP in pediatric patients with AN and obesity compared to patients ≤ the 90th percentile for weight, though AN severity degree was not analyzed. In our study, diastolic BP increased with an increase of AN severity degree in neck by Burke's scale [42]. Though the mechanism of increased BP in patients with AN is unknown, hypertension and obesity are strongly associated and can lead to cardiovascular disease and a higher risk of stroke, increasing the chance of early death or disability [43].

No statistically significant differences in fasting glucose concentrations were found between the groups in this study, which may be due to the higher fasting hyperinsulinism in participants with a higher increment of AN severity degree in neck by Burke's scale. The presence of hyperinsulinism in obese patients is usually associated with IR (measured as an increase in HOMA-IR) due to the increased insulin-like growth factor-1 (IGF-1) cell receptors in states of hyperinsulinemia. These receptors promote the epidermal growth factor receptor (EGFR), fibroblast growth factor receptor (FGFR), and the proliferation of epidermal keratinocytes and dermal fibroblasts when activated [44, 45]. Almost all of the participants in this study had IR, measured as a HOMA-IR concentration near the previously reported cutoff of 3.4 [46]. These results suggest that AN severity degree in neck by Burke's scale can be used as a clinical predictor of IR measured as increases in HOMA-IR, which contribute to alterations in WC, fasting insulin, triglycerides, and HDL-C, are considered cardiometabolic risk factors in obese children [47]. Similar to previous studies, we found that WC, triglycerides, total cholesterol, LDL-C, and HOMA-IR were increased [48, 49] and associated with the presence of AN [50, 51]. Specifically, we found that these variables were associated with grade 3 of AN severity degree in neck by Burke's scale (Table 2).

The presence of metabolic disorders is also represented by an inverse relationship between leptin and adiponectin concentrations in obese patients [52]. It is unclear why there is no correlation between WC, triglycerides, total cholesterol, LDL-C, or HOMA-IR with AN severity degree in neck by Burke's scale grade 4 in our study, though it may be due to increased leptin concentrations that play a compensatory role in patients with insulin intolerance or resistance due to more severe obesity. Leptin concentrations are higher in obese patients than in those of normal weight, and our study shows that leptin concentrations also increase with the severity of AN severity degree in neck by Burke's scale. When the mass of adipose tissue increases, the synthesis and secretion of leptin increase, stimulating a decrease in appetite, increase in energy expenditure, reduction in lipogenesis, and increase in lipolysis. Also, leptin upregulates proinflammatory cytokines such as interleukin 6, which is associated with dyslipidemias, IR, and type 2 diabetes [53]. In contrast, hyperinsulinemia [54] and IR are indirectly related to hyperleptinemia [55].

Adiponectin has anti-inflammatory properties and downregulates the expression and release of several proinflammatory immune mediators. A leptin/adiponectin imbalance may be an important risk factor of developing type 2 diabetes and cardiovascular diseases associated with abdominal obesity [52]. Adiponectin concentration has been reported to be a risk factor for the development of type 2 diabetes in children [56]. In addition, decreased adiponectin and increased IR are risk factors for metabolic syndrome [57]. The association of adiponectin and dyslipidemias is based on the fact that adiponectin exhibits its effects through the sensitivity of the body to insulin and the oxidation of fatty acids [58, 59] via the activation of several signaling molecules including adenosine monophosphate-activated protein kinase, p38-MAPK, JNK, the PPAR*γ* transcription factor, and NF-ƙ*β* in various tissues. These signals are transduced via the adiponectin receptor signaling pathway [60, 61] and may have a genetic component [62].

To our knowledge, this is the first study to report that AN severity degree in neck by Burke's scale grade 3 is related to metabolic changes in obese children, suggesting that AN severity degree in neck by Burke's scale can be used as a clinical predictor for IR and hypertriglyceridemia that allows for timely treatments through changes in lifestyle, supplements, or medications [63].

However, this study has some limitations as follows:(1) We recognize that our study group analyzed was small due to the type of sampling; however, there are no similar studies reported previously and it could be considered as a pilot, suggesting that it be carried out on a large scale in the future by increasing the size of the sample in order to determine sensitivity as a clinical tool of AN severity degree in neck by Burke's scale in obese children for screening for cardiometabolic alterations. Despite the size of the sample, our study showed statistically significant associations. (2) We did not perform an analysis of consumption frequency or 24 h dietary recall, which could infer the results; however, none of the included participants carried out any type of pharmacological or nonpharmacological treatment such as diet, physical activity [64], or lifestyle changes to reduce obesity. In addition, they did not know the presence of cardiometabolic alterations at the time of accepting their participation in the study. (3) Clinical and biochemical data related to changes in puberty and growth stages of the participants were not collected, which may influence the onset behavior of IR and AN severity degree [11]; although an association with the presence of early puberty mainly in women, it has not yet been completely clarified. (4) Our cross-sectional study only permits the establishment of associations of AN severity degree in neck by Burke's scale with cardiometabolic alteration but does not allow for the determination of causality. Efforts to clarify the causal relationship of the variables included in this study may yield new strategies according to screening approaches for obesity, IR, and related disorders. (5) Ancestry of the participants was not analyzed. It has been reported that patients with Native American ancestry have a genetic predisposition to dyslipidemia and the contribution to the presence of cardiometabolic alterations [65–67].

We assume that AN severity degree in neck by Burke's scale could be used as a screening biomarker to obesity complications in vulnerable children; however, future research is required to identify its application through interventions, aimed at preventive measures for screening and early detection of obesity complications, including insulin resistance, dyslipidemia, and impaired fasting glucose, as well as the development of specialized multidisciplinary teams responsible for implementing the findings.

## Figures and Tables

**Table 1:** General characteristics and clinical, anthropometric, metabolic, and inflammatory profiles by Burke's classification in obese children.

Burke's classification scale						
	0 (N=21, 22.10%)	1 (N=30, 31.57%)	2 (N=17, 17.89%)	3 (N=12, 12.63%)	4 (N=15, 15.78%)	^*∗*^ *p* value
Age (years)	9.52 ±1.6	9.0 ±1.8	8.76 ±2.01	9.33 ±1.92	9.33 ±1.44	0.74
Male (%)	19.67	37.7	19.67	9.84	13.12	0.33
Female (%)	26.47	20.59	14.71	17.64	20.59	

FHOB (%)					
No	21.57	39.22	13.73	11.76	13.73	0.44
Yes	26.09	21.74	21.74	13.04	17.39	

FHT2D (%)					
No	22.09	30.23	11.63	19.77	16.28	0.43
Yes	33.33	33.33	8.33	16.67	8.33	

BMI (kg/m^2^)	22.7 ± 2.8	23.9 ± 3	23.6 ± 3.3	25.3 ± 2.7	24.9 ± 2.4	0.76
Waist circumference (cm)	76.1 ± 8.2	80.3 ± 8.1	78.2 ± 11.4	83.0 ±9	80.9 ± 8	0.49
SBP (mmHg)	102.6 ± 9.6	102.8 ± 11.3	103.0 ± 12.6	109.6 ± 12.8	101.1 ± 9.3	0.63
DBP (mmHg)	67.5 ± 7.5	69.6 ± 9.5	68.1 ± 17.5	72.1 ±9.2	71.9 ± 8	0.001
Glucose (mg/dL)	87.5 ± 9.6	86.9 ± 6.3	86.4 ± 7.5	86.9 ± 8.4	83.4 ± 6.9	0.32
Insulin (µU/ml)	10.2 ± 5.6	9.0 ± 5.2	11.1 ± 6.9	13.9 ± 8.3	12.6 ± 5.7	0.15
HOMA-IR	2.1 ± 1.2	1.9 ± 1.2	2.4 ± 1.7	3.1 ± 2	2.7 ± 1.3	0.3
Total cholesterol (mg/dL)	139.0 ± 27	152.9 ± 35.7	136.4 ± 34.3	172.7 ± 41.3	153.8 ± 27.8	0.46
Triglycerides (mg/dL)	97.2 ± 38.9	102.7 ± 33.2	112.8 ± 49.4	149.7 ± 68.7	117.2 ± 49.3	0.02
HDL-C (mg/dL)	41.9 ± 9.9	45.4 ± 10.4	42.0 ± 9.2	42.1 ± 10.9	40.7 ± 7.8	0.8
LDL-C (mg/dL)	94.8 ± 21.2	105.3 ± 29	90.8 ± 25.3	122.9 ± 32.7	106.8 ± 24	0.47
Adiponectin (µg/mL)	12 ± 1.8	12.2 ± 2.2	12.4 ± 1.4	12.1 ± 1	12.1 ± 1.2	0.02
Leptin (ng/mL)	36.6 ± 12.2	38.8 ± 15.3	39.18 ± 12.3	37.7 ± 8.7	41.4 ± 9.4	0.18

DBP: Diastolic blood pressure; SBP: systolic blood pressure; BMI: body mass index; FHOB: family history of obesity; FHT2D: family history of type 2 diabetes. Results are shown as mean ± standard deviation.^*∗*^*p* < 0.05 indicates a statistical significance.

**Table 2:** Association of severity degree of an in neck by Burke's scale with WC, HOMA-IR, and lipid profile in obese children.

	Burke's scale of AN in neck	Coef.	Std. Err.	95% (CI)	*p* value
WC (cm)	1	4.73	1.81	(1.12; 8.32)	0.01
	2	2.90	2.7	(−1.24; 7.02)	0.17
	3	5.49	2.31	(0.88; 10.08)	0.02
	4	3.26	2.17	(−1.05; 7.58)	0.13

HOMA-IR	1	0.67	0.40	(−0.12; 1.48)	0.10
	2	0.92	0.52	(−0.11; 1.96)	0.08
	3	1.43	0.58	(0.27; 2.59)	0.01
	4	1.01	0.58	(−0.12; 2.16)	0.08

Triglycerides (mg/dL)	1	7.78	12.89	(−17.8; 33.42)	0.54
	2	18.89	14.61	(−10.17; 47.95)	0.20
	3	44.20	16.57	(11.24; 77.16)	0.009
	4	14.29	15.95	(−17.42; 46.01)	0.37

Total Cholesterol (mg/dL)	1	13.06	9.80	(−6.42; 32.56)	0.18
	2	-4.56	11.25	(−26.94; 17.80)	0.70
	3	29.51	12.28	(4.60; 54.42)	0.02
	4	10.38	12.28	(−14.05; 34.81)	0.40

LDL-C (mg/dL)	1	10.35	7.78	(−5.13; 25.83)	0.19
	2	−4.14	8.82	(−21.22; 13.24)	0.64
	3	26.94	10.10	(6.85; 47.03)	0.009
	4	11.07	9.73	(−8.28; 30.43)	0.25

Results were adjusted by sex, BMI, and age. Coef., coefficient; Std. Err., standard error; WC, waist circumference; HOMA-IR, homeostatic model of insulin resistance; LDL-C, low-density lipoprotein cholesterol; CI, confidence interval; AN, Acanthosis nigricans.

## Data Availability

Registration number R-2012-785-071 for data used to support the findings of this study may be released upon application to the Research and Bioethics Committee of the Instituto Mexicano del Seguro Social (Mexican Social Security Institute) that can be contacted at COMITÉ DE ÉTICA EN INVESTIGACIÓN DEL IMSS: Avenida Cuauhtémoc 330 4° piso Bloque “B” de la Unidad de Congresos, Colonia Doctores. México, D.F., CP 06720. Teléfono (55) 56276900 extensión 21216 en horarios de 9 a 16 horas de lunes a viernes, Correo electrónico: comiteeticainv.imss@gmail.com.

## Abbreviations

AN: Acanthosis nigricans
WC: Waist circumference
HOMA-IR: Homeostatic model assessment for insulin resistance
IR: Insulin resistance
GI: Glucose intolerance
IMSS: Mexican Institute of Social Security
BMI: Body mass index
BP: Blood pressure
LDL-C: Low-density lipoprotein cholesterol
HDL-C: High-density lipoprotein cholesterol
IGF-1: Insulin-like growth factor-1
EGFR: Epidermal growth factor receptor
FGRF: Fibroblast growth factor receptor
DBP: Diastolic blood pressure
SBP: Systolic blood pressure
FHOB: Family history of obesity
FHT2D: Family history of type 2 diabetes.
